# Dose–response study of a fenugreek-based antibiotic alternative in Bábolna Tetra-SL chicks (1–42 days old) with mixed bacterial infections

**DOI:** 10.3389/fvets.2025.1570387

**Published:** 2025-04-24

**Authors:** Ádám Kerek, Ábel Szabó, Péter Ferenc Dobra, Krisztina Bárdos, Bettina Paszerbovics, Zsófia Bata, Viviána Molnár-Nagy, Ákos Jerzsele, László Ózsvári

**Affiliations:** ^1^Department of Pharmacology and Toxicology, University of Veterinary Medicine, Budapest, Hungary; ^2^National Laboratory of Infectious Animal Diseases, Antimicrobial Resistance, Veterinary Public Health and Food Chain Safety, University of Veterinary Medicine, Budapest, Hungary; ^3^Department of Pathology, University of Veterinary Medicine, Budapest, Hungary; ^4^Department of Veterinary Forensics and Economics, Institute of Economics and Biostatistics, University of Veterinary Medicine, Budapest, Hungary; ^5^Department of Biostatistics, Institute of Economics and Biostatistics, University of Veterinary Medicine, Budapest, Hungary; ^6^Dr. Bata Zrt., Ócsa, Hungary

**Keywords:** *Salmonella enterica*, *Escherichia coli*, antibiotic alternatives, laying hens, *Trigonella foenum-graecum*, fenugreek, poultry, AMR

## Abstract

**Introduction:**

Combating antimicrobial resistance is one of the most pressing public health challenges of our time. The rapid spread of resistant, zoonotic bacterial strains in livestock farming is increasingly raising concerns about the need to reduce antibiotic use. Because of this, there is an urgent need for safe and effective alternatives in animal husbandry.

**Methods:**

This study aimed to perform an *in vivo* the dose–response analysis of fenugreek (*Trigonella foenum-graecum*), as a plant-based antibiotic alternative feed supplement in Bábolna Tetra-SL chicks (1–42 days old) with a 1:1 sex ratio. A total of 270 chicks were randomly assigned to 18 groups (15 birds per group) and subjected to six different treatment groups in three replicates: fenugreek at 1×, 10×, and 100× doses, an antibiotic-treated group (enrofloxacin), a positive control group (infection only), and a negative control group (no infection or treatment). The infection was induced using mixed *Salmonella Enteritidis* and *Escherichia coli*, administered via gavage on days 3 and 4 of life. The birds were monitored for clinical symptoms, body weight, feed intake, and *Salmonella* shedding through cloacal swab samples. Statistical analyses included mixed-effect logistic regression for mortality, mixed-effect linear models for weight gain, two-way ANOVA for feed efficiency, and random effects continuation ratio models for *Salmonella* isolation.

**Results:**

Significant interactions for Group:Day and Sex:Day in weight gain were identified (*p* < 0.0001 for both). Additionally, the 1 × dose group showed significantly reduced *Salmonella* shedding compared to the positive control group on day 33 (*p* = 0.0031). The low-dose group (1×) demonstrated the most promising results, showing a 63% reduction in *Salmonella* shedding on day 10 and 31% on day 17. This group exhibited the fewest clinical symptoms, no diarrhea, and the lowest individual and specific feed intake up to day 24.

**Discussion:**

The findings of this study suggest that low-dose fenugreek supplementation could be a viable strategy for reducing *Salmonella* shedding in poultry, potentially contributing to reduced antibiotic use in poultry farming and thus playing a role in the global effort to combat antimicrobial resistance. Future research will involve large-scale industrial trials and next-generation sequencing to evaluate the additive’s impact on gut microbiota composition.

## 1 Introduction

The foundation of modern medicine was laid by antibiotics, which became so pivotal that the mid-20th century is often referred to as the “antibiotic era.” It was once believed that infectious diseases would be eradicated by the end of the century. However, this expectation has not materialized. Instead, the increasing emergence of multidrug-resistant (MDR) bacterial strains has become a critical global health threat (1).

In livestock farming, the use of antibiotics at subtherapeutic doses has been a long-standing practice to sustain and enhance production performance, taking advantage of their growth-promoting properties (2). Unfortunately, the excessive and improper use of antibiotics has become a key driver of antimicrobial resistance (AMR) among food-producing animals. It has also contributed to the emergence of resistant bacteria responsible for foodborne diseases, which carry significant public health risks (3). Addressing these concerns requires developing safe and effective feed additives that can maintain livestock productivity while reducing the spread of drug-resistant pathogens (4). This issue is particularly pressing in the poultry industry (5), which is the second-largest consumer of antibiotics after the swine industry (6).

Alternative solutions should be explored to replace antibiotics, such as antimicrobial peptides (7), various plant-based essential oils (8) and extracts (9, 10), or even the use of propolis (11–13), medium-chain fatty acids and triglycerides (14). Additionally, the application of appropriate pharmacokinetic/pharmacodynamic (PK/PD) models is essential for optimizing treatment regimens (15). Proper biosecurity measures on livestock farms significantly contribute to reducing the risk of infections, thereby minimizing the unnecessary use of antibiotics (16, 17).

Among the diseases affecting livestock, zoonotic diseases hold exceptional significance. Beyond their economic impact—such as reduced productivity, stock loss, and increased veterinary costs—they can directly affect human health and pose a global threat. Among zoonotic diseases, salmonellosis stands out due to its prevalence. While its spread has declined significantly thanks to strict regulations, it remains one of the leading causes of foodborne illnesses worldwide. According to some estimates, salmonellosis is responsible for over 90 million cases of diarrhea-related symptoms annually (18). The severity of the disease is underscored by the fact that it causes more than 150,000 deaths per year, with children under the age of four being most at risk of fatal outcomes (19). The genus *Salmonella* comprises rod-shaped, Gram-negative, facultative anaerobic bacteria, with *S. enterica* recognized as the most significant pathogenic species including over 2,600 distinct serotypes (20). Approximately 99% of the *Salmonella* strains causing infections in humans and other mammals belong to this species. Infection is typically characterized by symptoms such as weakness, fever, and acute enteritis, often accompanied by diarrhea of varying severity. Symptoms generally appear 6–72 h after colonization, usually requiring the ingestion of more than 50,000 bacteria, most commonly through contaminated food or water. In most cases, symptoms subside and resolve within 5–7 days without treatment. However, severe complications may occur in some instances (21).

In developed countries, the intestinal tract of food-producing animals is considered the primary reservoir of *Salmonella* strains, which can easily lead to contamination of food products (22). One major source of *Salmonella* infection in humans is the consumption of contaminated poultry meat, highlighting the critical role of poultry farming and processing in the transmission of infections. Thus, without the protection that antibiotics once afforded, as global demand for meat and poultry products continues to rise, the risk of *S. enterica* infections also increases (23). The second source of *Salmonella* infection from poultry is caused by the improper management of manure and bedding materials. The application of poultry manure to agricultural land is a widespread practice due to its accessibility and lower cost compared to commercial fertilizers. However, without adequate treatment, this practice poses a high risk due to the potential contamination with enteric pathogens (*S. enterica*, *E. coli*, *Campylobacter jejuni*). Transfer to crops can result in the contamination of vegetables and fruits consumed by humans, as well as feed crops, perpetuating the circulation of these bacteria in the environment (24).

For centuries, humanity has utilized various plants, extracts, and preparations in medicine, and recent decades have seen a surge in publications scientifically validating their effectiveness (34). With industrialization and modern medicine, the use of medicinal plants and their applications fell into relative obscurity. However, the emergence of new technologies — capable of overcoming processing challenges and significantly standardizing plant-based products — has reignited interest in their pharmaceutical applications. This renewed focus is driven in part by environmental pressures such as antimicrobial resistance (AMR) and increasing consumer demand for natural, plant-derived compounds (25).

Fenugreek (*Trigonella foenum-graecum*) is renowned for its numerous beneficial properties and has long been a staple of traditional medicine in Asia and Mediterranean countries. It contains a variety of bioactive compounds, including alkaloids, tannins, flavonoids, glycosides, and terpenoids, which contribute to its antimicrobial and anti-inflammatory activities (26). Supplementing broiler chicken feed with fenugreek seed powder (1–1.5%) has been shown to improve feed conversion efficiency and increase final body weight in birds (27). In laying hens, it enhanced feed intake and positively influenced yolk color, particularly during the second production cycle (28). Fenugreek also benefits the immune system and gut microbiome composition of poultry, likely by modulating the interaction between these systems, making it a natural, health-promoting feed additive suitable for use in poultry farming (29).

Following the World Health Organization’s recommendations to minimize the use of critically important antimicrobials in human medicine (30). This study evaluates the dose–response effects of a fenugreek-based feed additive in broiler chickens. Using a conventional *in vivo* trial, we assess its potential as an antibiotic alternative by examining performance indicators and its efficacy in mitigating a combined *S. enterica* and *E. coli* infection.

## 2 Materials and methods

### 2.1 Origin of birds, immune status, and groups

The study was conducted with a total of 270 Bábolna Tetra-SL laying hybrid chicks, with a 1:1 sex ratio. The inclusion of both male and female chicks was necessary to evaluate the general efficacy of the fenugreek-based antibiotic alternative feed supplement on mixed-sex populations, ensuring broader applicability of the results beyond female-only studies. The primary focus of this study was to assess overall health indicators, such as mortality, clinical symptoms, weight gain, and *Salmonella* shedding. While weight gain was analyzed with sex-specific statistical methods, other outcomes (e.g., mortality, pathology) were not analyzed separately by sex, as the primary goal was to establish overall efficacy and identify the most effective dose.

Birds were obtained from the Szarka Ferenc Hatchery of Bábolna Tetra Kft. At the hatchery, the day-old chicks were vaccinated with NOBILIS^®^ RISMAVAC + CA126 (Marek’s disease), Nobilis ND C2 (Newcastle disease), and Poulvac IB Primer (infectious bronchitis) vaccines. The bird trial, classified as mild in severity (license number: PE/EA/01174–6/2023), involved feeding the plant-based test material mixed into the feed. The composition of the basal diet fed to the chickens is detailed in Tables 1, 2. The composition of the premix is provided in Supplementary Table S1.

**Table 1:** Detailed composition of the pre-starter and starter feed.

Ingredient	Pre-starter	Starter
Wheat	40.0%	40.0%
Soy	29.2%	31.2%
Corn	24.8%	23.8%
Sunflower seed oil	2.0%	1.0%
Premix	4.0%	4.0%

**Table 2:** Composition of the basic feed fed in the animal experiment during two-phase feeding.

Feed			Pre-starter	Starter
Nutrients			0–3 weeks	4–6 weeks
Metabolizable energy		MJ/kg	12.35	12
Metabolizable energy		kcal/kg	2,950	2,870
Crude protein		%	20	18
Total amino acids	Lysine	%	1.2	1
	Methionine	%	0.48	0.42
	Methionine + cystine	%	0.84	0.74
	Threonine	%	0.75	0.65
	Valine	%	0.93	0.78
	Arginine	%	1.22	1.02
	Tryptophan	%	0.24	0.22
	Isoleucine	%	0.84	0.75
Digestible amino acid	Lysine	%	1	0.83
	Methionine	%	0.4	0.35
	Methionine + cystine	%	0.7	0.6
	Threonine	%	0.63	0.55
	Valine	%	0.76	0.65
	Arginine	%	1.02	0.84
	Tryptophan	%	0.2	0.18
	Isoleucine	%	0.69	0.62
	Linoleic acid	%	1.5	1.25
Calcium		%	1	1
Phosphorus (available)		%	0.48	0.44
Sodium		%	0.17	0.17
Chloride		%	0.18	0.18

Upon arrival, the birds were randomly assigned to 18 groups, with 15 birds per group. The experiment included six treatment groups examined in three replicates: the test substance at a 1 × dose (0.1 g/kg), 10 × dose (1 g/kg), and 100 × dose (10 g/kg), along with an antibiotic-treated group, a positive control group (infection only), and a negative control group (no infection or treatment). The use of three replicates per treatment group aligns with the European Food Safety Authority (EFSA) guidelines, which recommend a minimum of three independent *in vivo* studies to demonstrate the efficacy of feed additives (31). This approach is considered sufficient to provide statistical robustness in evaluating the efficacy of feed additives. The exact group allocations are summarized in Supplementary Table S2. To ensure reliable identification, each bird was tagged with a unique number (1–270). The components of the test material fed to the birds are listed in Supplementary Table S3.

### 2.2 Infection and monitoring

Infection was induced with *Salmonella Enteritidis* and *E. coli*. The strains were prepared by streaking onto tryptic soy agar (Biolab Zrt., Budapest, Hungary) and incubated at 37°C for 24 h. The following day, 150 mL of pre-warmed tryptic soy broth (TSB) at 37°C was inoculated with 18–20 colony-forming units (CFU) of each strain using a sterile inoculating loop, followed by incubation at 37°C for 4 h. The method, based on prior pilot studies, ensured achieving a concentration of at least 10^8^ CFU/mL for both strains during their exponential growth phase. The mixed infection was administered to birds 1–225 on the 3rd day of life and repeated on the 4th day, using a gavage tube to deliver 0.5 mL of *Salmonella Enteritidis* and 0.5 mL of *E. coli* suspension to each bird. For the antibiotic group, treatment with enrofloxacin (10 mg/kg body weight mixed into drinking water for 5 days) was started the day after the second infection and continued for 5 days.

The birds were monitored daily for clinical symptoms, including changes in fecal consistency, presence of bloody stool, fecal contamination around the cloaca, urates deposition near the cloaca, lameness, wing drooping, and lethargy. The body weight of deceased birds was recorded, and the carcasses were sent to the Department of Pathology of University of Veterinary Medicine (UVMB) for necropsy to determine the cause of death.

On the second day post-infection, five birds from each group were randomly selected and euthanized for necropsy at the Department of Pathology of UVMB, in order to examine early lesions indicative of salmonellosis that would no longer be detectable by the sixth week of life.

### 2.3 *Salmonella* isolation

On the pre-determined sampling days, cloacal swab samples were enriched by immersing them in Rappaport Vassiliadis broth (Biolab Zrt., Budapest, Hungary) – 3 mL/tube – and incubating at 41°C for 24 h. Subsequently, 50 μL of each sample was streaked onto Rambach agar (CheBio Fejlesztő Kft., Budapest, Hungary) and incubated at 41°C for another 24 h. The presence or absence of *Salmonella* was assessed based on growth characteristics. In ambiguous cases, additional plating was performed on Xylose lysine deoxycholate (XLD) (Biolab Zrt., Budapest, Hungary) and *Salmonella*-selective agars (Biolab Zrt., Budapest, Hungary) for confirmation.

### 2.4 Measurement of body weight and feed consumption

Body weight was individually measured weekly (D0, D7, D14, D21, D28, D35, D42) using a scale with gram-level precision. Each bird was tagged with a unique identification number to ensure accurate data attribution.

Daily feed consumption was recorded by groups using back-weighting on Mondays, Wednesdays, and Fridays. For this purpose, feed was provided in measured quantities the morning before each weighing: 0.5 kg per group during weeks 1–3 and 1 kg per group during weeks 4–6. The remaining feed was weighed the following morning, and consumption was calculated as the difference between the initial feed quantity and the remaining feed.

On the final day of the experiment (D42), the birds were humanely euthanized using Euthasol 40% Injection A.U.V. at a dose of 100–200 mg/kg body weight. The injection was administered intravenously into the wing vein following appropriate restraint. This method induces full anesthesia before paralyzing the respiratory center, ensuring a completely pain-free process.

Subsequently, the birds were transferred to the Department of Pathology of UVMB, where all specimens underwent macroscopic examination. Additionally, histopathological analysis was conducted on two birds per group.

### 2.5 Statistical analysis

At the end of the experiment, the summarized data were statistically evaluated by the Department of Biostatistics of UVMB using R software version 4.3.2. Isolation results (ordinal data from 0 to 3) were analyzed using a random effects continuation ratio model, with treatment groups and sampling days as explanatory variables and chicken ID as a random effect to account for repeated measurements. Group comparisons were conducted using the emmeans() and pairs() functions, with multiplicity correction via the Dunnett method (32).

Weight gain analysis involved log-transforming body weight data to linearize its relationship with time. A linear mixed-effects model was fitted, including fixed effects for baseline weight, treatment group, sex, and day, with random intercepts for individual birds. Pairwise comparisons were performed with emmeans() and pairs(), applying the Dunnett correction (33).

Feed efficiency was examined through two-way ANOVA, with treatment group and week as explanatory variables (34). Mortality analysis used a mixed-effect logistic regression model with treatment group and date as fixed effects and cage effect as a random effect. The lme4 package and glmer() function were used, applying the Dunnett correction for multiple comparisons (35).

Feed consumption was analyzed with a linear model using the gls() function, with day and treatment group as fixed effects. Variance structures were specified by cage, and correlation structures accounted for the repeated measures over time (33, 36).

## 3 Results

### 3.1 Doses used for infection

To determine the colony-forming unit (CFU) count used during the infection, a tenfold serial dilution of the bacterial suspension was prepared prior to the infection. From each dilution, 50 μL was plated onto Petri dishes, followed by 24 h of incubation. After incubation, the plates were evaluated, and the CFU count was based on the Petri dish containing the smallest, countable number of bacterial colonies. This count was then multiplied by the dilution factor associated with the plate and multiplied by 20 as 50 μL was used for plating. Table 3 presents the CFU counts administered via crop gavage during the experiment (0.5 mL).

**Table 3:** The number of colony-forming units (CFUs) inoculated during infections, categorized by bacterial species.

Strains	First infection (0.5 mL)	Second infection (0.5 mL)
*Salmonella enterica* 204/22	2.5 × 10^8^	1.9 × 10^9^
*Escherichia coli* 762/22	1.25 × 10^9^	5 × 10^10^

### 3.2 Clinical symptoms and mortality analysis

In the days following the infection, the group treated with the 1 × dose group exhibited the fewest clinical symptoms, with minimal changes observed in fecal consistency. The group treated with the 10 × dose group showed slightly softer feces, occasionally with minor blood traces. Severe symptoms, including watery diarrhea and lethargy, were most pronounced in the 100 × dose and positive control groups. Diarrhea was less severe in the antibiotic-treated group, and no symptoms were observed in the negative control group.

**Figure 1:**
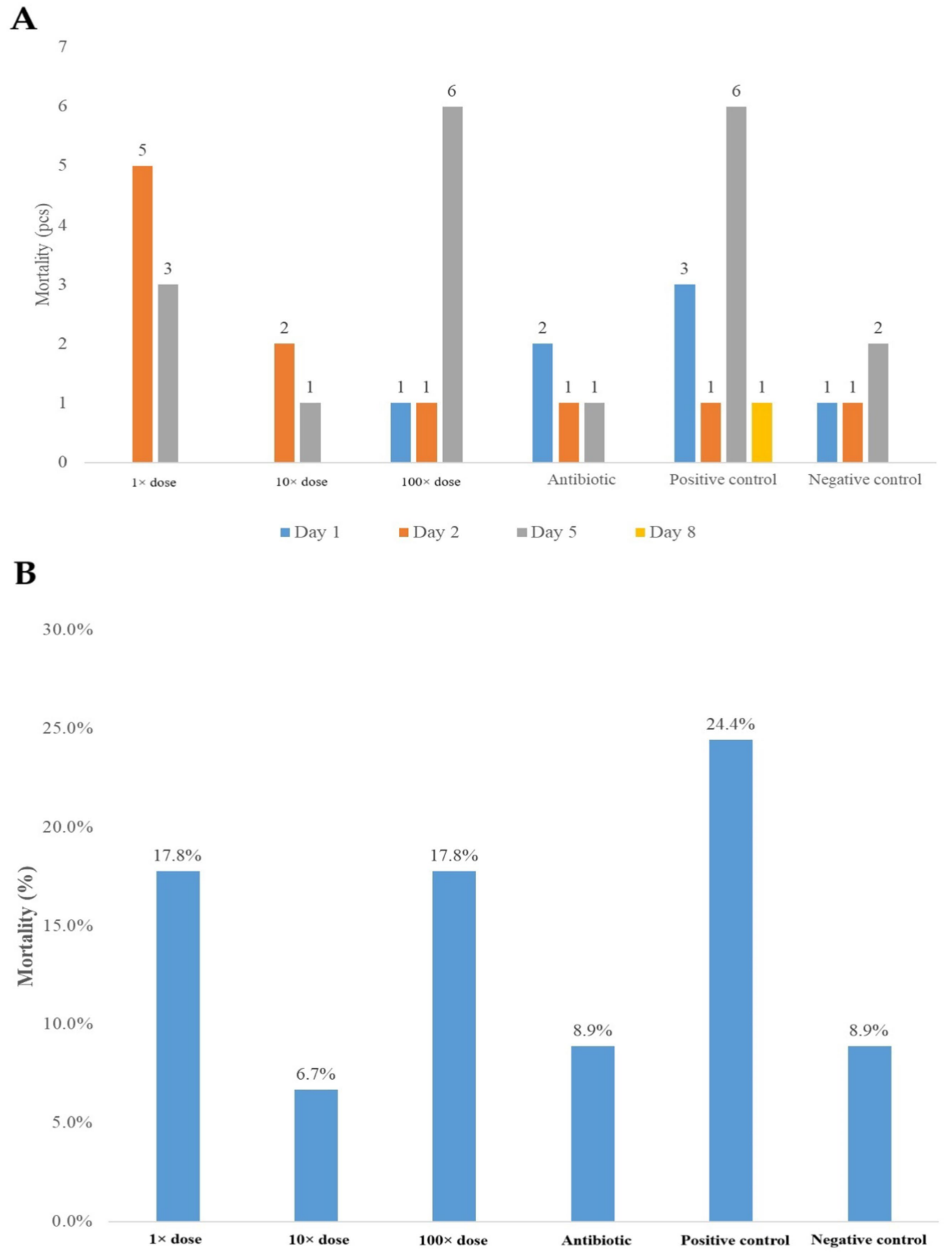
**(A)** The progression of mortalities in each group at different time points following the infection. **(B)** The percentage mortality in each group relative to the total group size following the infection.

Following the second infection (D4), mortality occurred in all groups, peaking on the third day post-infection in the positive control and 100 × dose groups. Figure 1A shows the number of deceased birds by time of death, while Figure 1B illustrates percentage mortality per group.

Mixed-effect logistic regression showed that the 10 × dose group had the lowest odds of mortality compared to the positive control group (0.262), but without statistical significance. The other groups also displayed reduced mortality odds, though not statistically significant (Supplementary Figure S1 and Supplementary Table S4).

**Figure 2:**
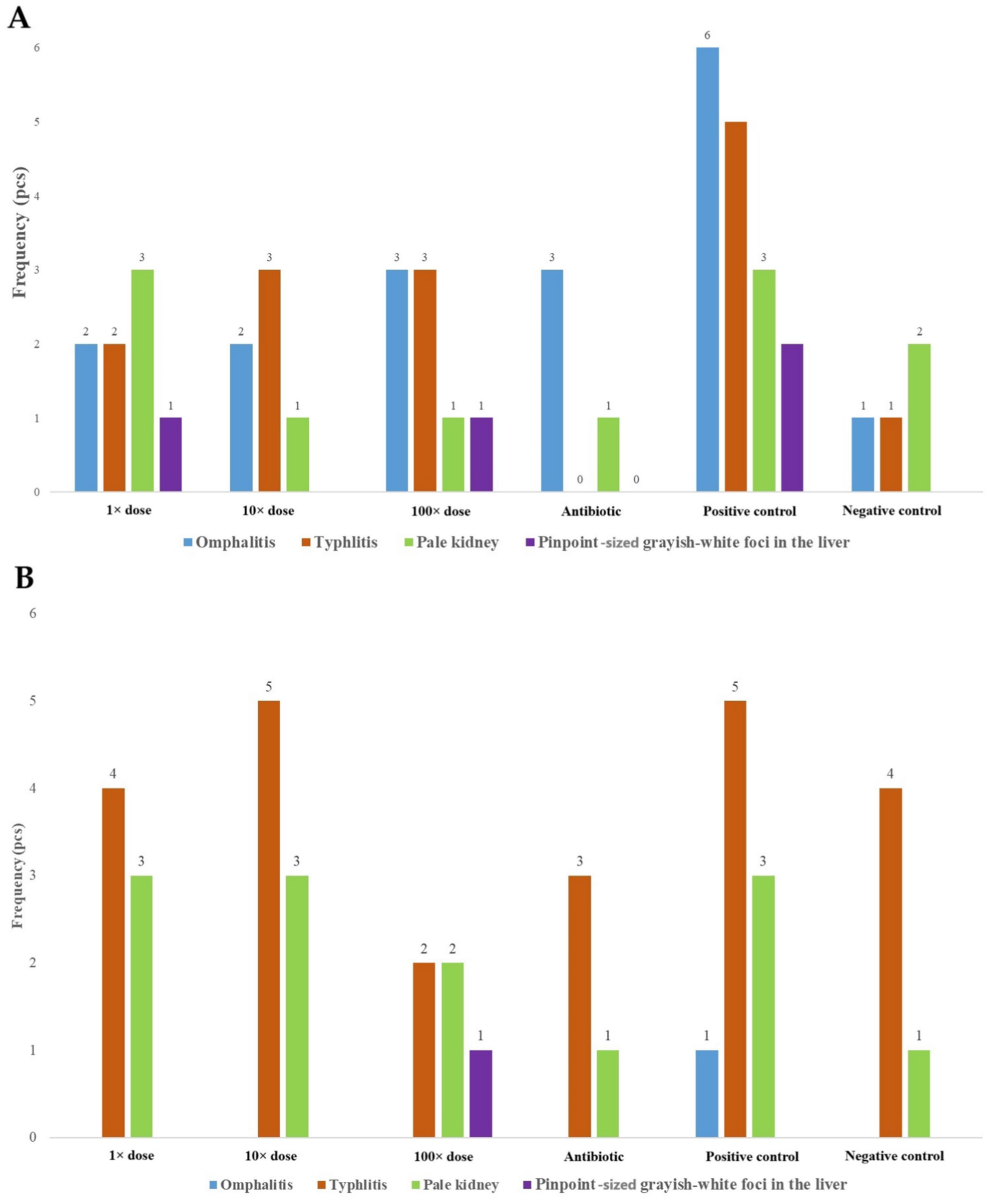
**(A)** The occurrence of key pathological findings observed during post-mortem examinations of deceased birds, categorized by treatment groups at day-old age. **(B)** The most common pathological findings observed during post-mortem examinations of euthanized birds at day-old age.

Post-mortem examinations revealed omphalitis in all groups except the antibiotic-treated and negative control groups. Typhlitis was most prevalent in the positive control group and present in all groups except the negative control. Symptoms such as pale kidneys and liver lesions indicative of septicemia were observed primarily in the 1 × dose, positive control, and 100 × dose groups (Figures 2A,B).

### 3.3 Body weight trends

**Figure 3:**
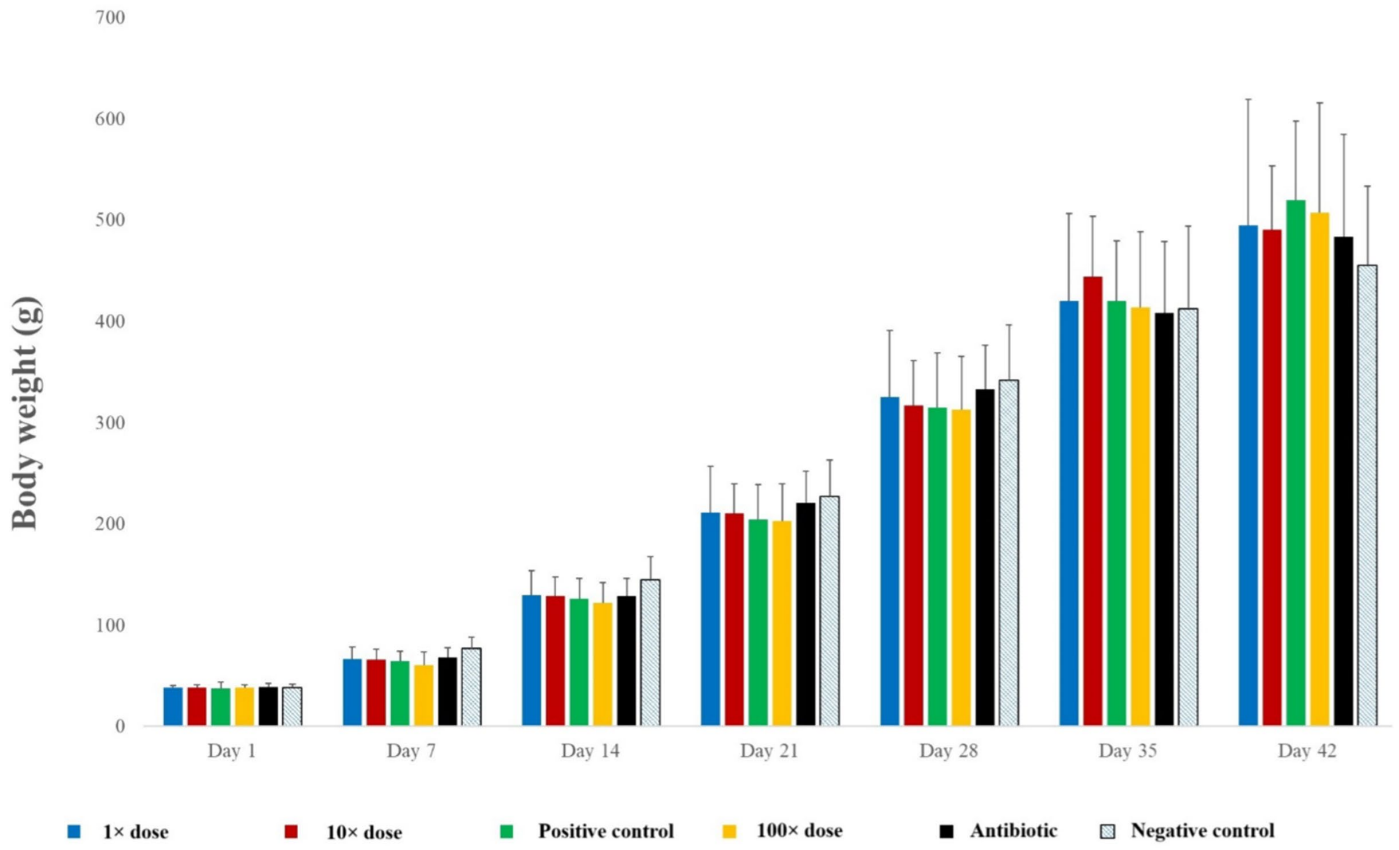
Weight gain trends by group across the individual measurement weeks.

The negative control group generally showed the highest weight gain until week four. From week five, the 10 × dose group showed the highest average weight gain, while the positive control and 100 × dose groups exhibited the largest gains by week six (Figure 3).

The mixed-effect linear model showed significant interactions for Group:Day (*p* < 0.0001) and Sex:Day (*p* < 0.0001), indicating that the effect of the treatment group on weight gain varies across different days, and the effect of sex on weight gain also varies across different days (Supplementary Table S5).

Each treatment group was compared to the positive control group on individual days. Significant differences were observed only between the negative and positive control groups on D7 (*p* = 0.0009), D14 (*p* = 0.0094), and D42 (*p* = 0.0106). All values are summarized in Supplementary Table S6.

We also evaluated the effect of sex on weight gain (Supplementary Table S7). A significance level of 0.05 was used, with multiplicity correction applied using the Tukey method. Significant differences were observed on D14 (*p* = 0.0054), D21 (*p* = 0.0009), D28 (*p* < 0.0001), D35 (*p* < 0.0001), and D42 (*p* < 0.0001).

### 3.4 Feed intake and feed conversion ratio (FCR)

**Figure 4:**
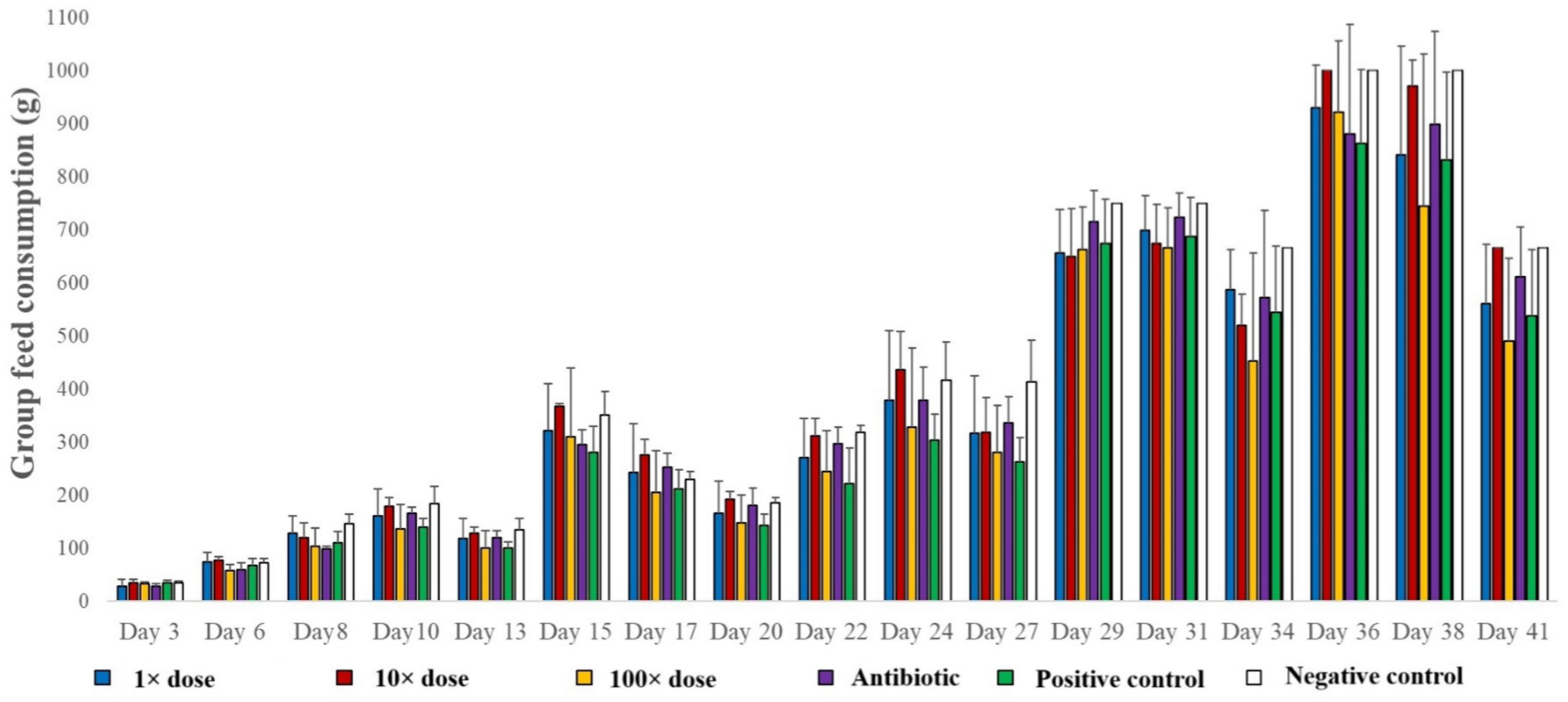
Feed consumption per group expressed in grams on each measurement day.

Feed consumption was calculated on both a group and individual basis to account for varying mortality rates. Significant feed wastage from scratching limited reliable measurements to the first 3 weeks (Figure 4). No statistically significant differences in feed intake were found between groups (Supplementary Table S8).

To calculate the feed conversion ratio (FCR), the ratio of the daily feed consumption per individual to the daily weight gain was used as a basis (Table 4). However, due to significant feed wastage caused by scratching, the FCR values calculated for the last 3 weeks could not be considered reliable. Although no significant differences were detected in the applied statistical model, the estimated average feed conversion ratio over the 4-week period was lowest in the 1 × dose group (Supplementary Table S9).

**Table 4:** Feed conversion ratio (FCR) trends by treatment group across weeks.

Week	1 × dose	10 × dose	100 × dose	Antibiotic	Positive control	Negative control
1	1.26	1.46	1.50	1.06	1.28	0.99
2	1.08	1.44	1.14	1.30	1.27	1.29
3	1.16	1.67	1.28	1.27	1.42	1.49

### 3.5 *Salmonella* isolation

**Figure 5:**
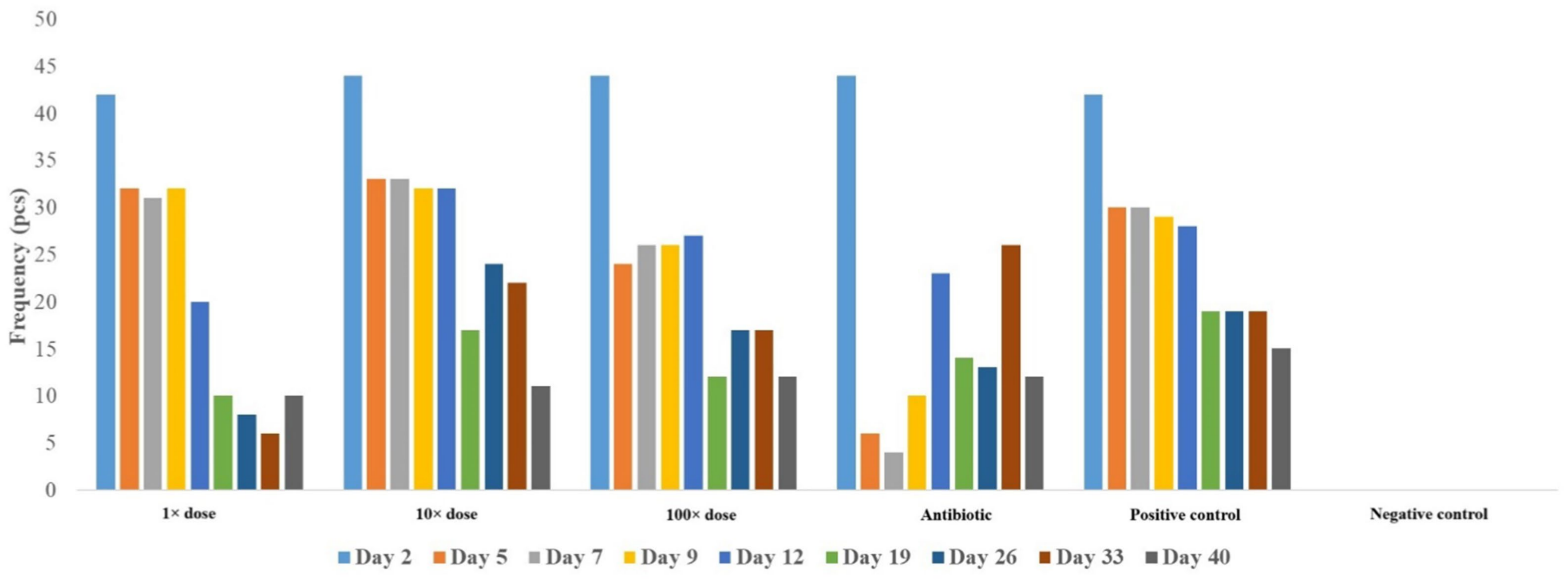
The number of *Salmonella*-shedding birds at each sampling point, by treatment group.

Following infection, *Salmonella* shedding persisted across all infected groups. Figure 5 shows the number of *Salmonella*-shedding birds in each group at the various sampling points. Over time, the most significant reduction in the number of shedding birds was observed in the 1 × dose-treated group. In the antibiotic-treated group, a sharp decrease in shedding birds was noted following treatment; however, complete eradication of *Salmonella* was not achieved, with intermittent shedding observed between sampling points.

The statistical analysis of *Salmonella* isolation data revealed significant differences on D5 and D7 (*p* < 0.0001; *p* = 0.0018) between the antibiotic-treated and positive control groups, indicating less severe *Salmonella* shedding in the antibiotic-treated group. Similarly, the 1 × dose group showed significantly reduced *Salmonella* shedding compared to the positive control group on D33 (*p* = 0.0031) (Supplementary Table S10). Although no other statistically significant differences were observed, the differences between the treated groups and the positive control group can be examined in Supplementary Table S10 by analyzing the ratio (odds ratio) column. This value indicates the odds ratio between the two groups. Values close to 1 suggest that the examined group and the positive control group yield similar results, deviations from 1 indicate potential differences between the groups in terms of *Salmonella* shedding. If the ratio is less than 1, it indicates that in the positive control group, the odds of receiving a specific score versus higher scores (e.g., the odds of receiving a score of 1 versus 2 or 3) for *Salmonella* isolation is higher than in the comparable group. Conversely, if the ratio is greater than 1, it suggests that the odds of specific score versus higher scores is higher in the comparable group than in the positive control group.

### 3.6 Pathology and histopathology results

**Figure 6:**
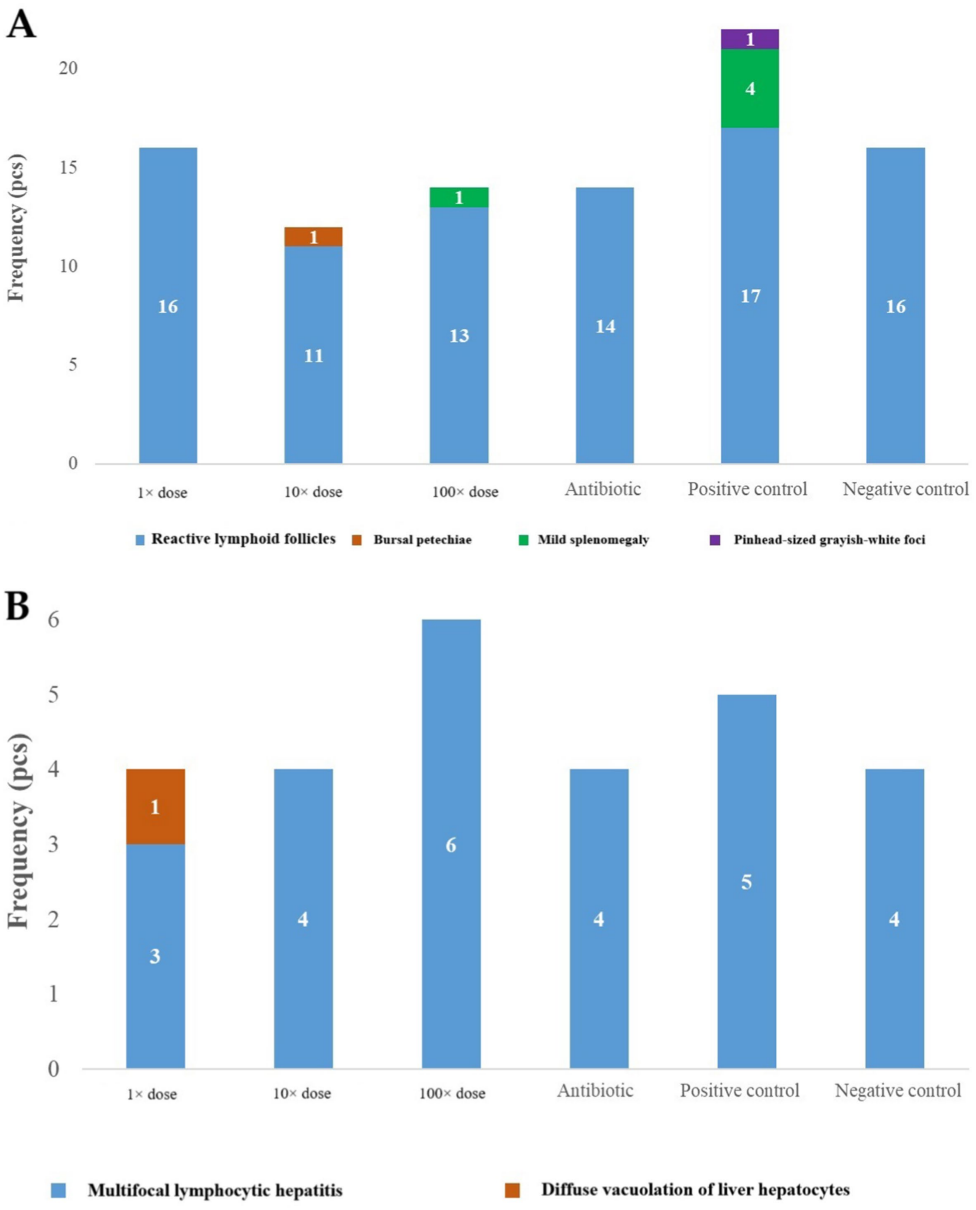
**(A)** The most significant gross pathological changes by treatment group at the end of the experiment. **(B)** The most significant histopathological changes by treatment group at the end of the experiment.

At the end of the experiment, the gross pathology examinations for all birds and histopathological analyses for two birds per group were conducted by the Department of Pathology of UVMB. The macroscopic lesions observed during the necropsies are summarized in Figure 6A. The most frequent findings included reactive lymphoid follicles in the intestines, splenomegaly, pinhead-sized grayish-white foci on the liver, and petechial hemorrhages in the bursa. The majority of these lesions were observed in the positive control group.

The results of the histopathological analysis of liver samples, conducted on a group-wise basis, are summarized in Figure 6B. Focal or multifocal lymphocytic infiltration, as well as diffuse vacuolization of hepatocytes, were noted. The highest number of pathological changes was observed in birds treated with the 100 × dose. However, compared to the negative control group, neither post-mortem examination nor histopathology revealed significant differences in any of the treated groups.

### 3.7 Summary of key findings and biological relevance

To provide a clearer overview of the most important findings, the key results from various analyses are summarized in Table 5. The table includes statistical significance, as well as a discussion of the potential biological relevance of each finding.

**Table 5:** Summary of key findings and biological relevance.

Parameter	Findings	Biological relevance
Mortality (odds ratio vs. positive control)	Lowest odds in 10 × dose group (OR: 0.262, not significant).	Although not statistically significant, the 10 × dose group showed the lowest mortality, suggesting potential protective effects.
Weight gain (negative control vs. positive control)	Significant differences on D7 (*p* = 0.0009), D14 (*p* = 0.0094), D42 (*p* = 0.0106).	Weight gain differences indicate that the negative control group generally performed better than the positive control group, suggesting detrimental effects of infection.
Weight gain (sex differences)	Significant differences on D14, D21, D28, D35, D42 (*p* < 0.0001).	Sex-based differences suggest the need for separate consideration of male and female birds in future studies, particularly in the context of weight gain and growth.
Feed conversion ratio (1 × dose group)	Lowest FCR in 1 × dose group over 4-week period (not statistically significant).	Improved feed efficiency in the 1 × dose group is promising for practical applications in poultry farming, potentially enhancing productivity with minimal input.
*Salmonella* shedding (1 × dose group vs. positive control)	Significant reduction in shedding on D33 (*p* = 0.0031).	The 1 × dose group demonstrated effective reduction in *Salmonella* shedding, indicating potential utility as an antibiotic alternative.
*Salmonella* shedding (antibiotic group vs. positive control)	Significant reduction in shedding on D5 (*p* < 0.0001), D7 (*p* = 0.0018).	While antibiotic treatment reduced shedding effectively, complete eradication was not achieved, highlighting limitations of conventional approaches.

## 4 Discussion

The infection was performed at 3–4 days old because chicks are most susceptible to infection during this early age and later attempts to induce infection are less likely to produce clinical symptoms, making it challenging to evaluate treatment efficacy. Regarding mortality, the highest death rate occurred on the day following the first infection in the positive control group. However, after the second infection, the highest mortality rate was observed in the 1 × dose group. On D5 post-infection, the greatest mortality was recorded in the positive control and the 100 × dose-treated groups. Overall, the positive control group experienced the highest total mortality rate (24.4%), followed by the 1 × dose and 100 × dose groups, both with 17.8% mortality. The mortality rates effectively reflect biological variability and clearly indicate the success of the infection. The treatment with the 10 × dose was the most effective in reducing mortality, which can be attributed to the compound’s ability to lower the likelihood of systemic establishment of the infection. This finding is consistent with previous studies demonstrating that probiotics such as *Bacillus toyonensis* can also improve growth performance and reduce mortality by modulating the gut microbiome and enhancing immunity (37). This could be attributed to the potential of the fenugreek extract to reduce *Salmonella*’s ability to colonize, adhere, and invade. Additionally, the observed effects may be influenced by alterations in the composition of the gut microbiome (38–40). This may be attributed to the bioactive compounds of fenugreek, such as diosgenin and saponins (39, 41).

Clinical symptoms were least pronounced in the 1 × dose group among the infected groups, while the 10 × dose group showed slightly more evident symptoms. In contrast, the positive control and 100 × dose-treated groups exhibited significant clinical signs, including severe diarrhea, bloody feces, urate deposits around the cloaca, and lethargy. Regarding clinical symptoms, the lower doses of the test material effectively reduced the likelihood of manifesting symptoms. However, as the concentration increased, the inverse effect was observed. This may be attributed to potential dose-dependent toxicity or saturation effects associated with higher concentrations of fenugreek. Excessive amounts of bioactive compounds, particularly saponins, may cause intestinal irritation or disrupt the gut microbiome balance, leading to negative health outcomes (29, 42–44). Interestingly, similar findings have been reported for other plant-based antimicrobials, where high concentrations can disrupt microbial balance, as observed with laurel leaf extracts (45).

Furthermore, high doses of fenugreek may induce pro-oxidative stress instead of providing antioxidant benefits, especially when exceeding the optimal threshold for effective biological activity (46–48). Additionally, such high doses could interfere with nutrient absorption mechanisms, reducing overall feed efficiency. Further studies focusing on microbiome analysis and dose–response mechanisms are needed to clarify these findings. The potential of plant-derived phytochemicals to modulate microbial balance and enhance host immune response has been demonstrated in several studies, including those targeting zoonotic infections such as giardiasis (49).

Among the deceased individuals, the majority of pathological findings were observed in the positive control group. Evidence of successful *Salmonella* infection (establishment) was indicated by typhlitis (50, 51), which was most pronounced in the positive control group, followed by the groups treated with 10 × dose and 100 × dose. Pale kidneys were most frequently observed in the positive control group and the 1 × dose group. Signs of systemic infection were suggested by the presence of pinpoint grayish-white foci in the liver, which were most commonly seen in the positive control group but were also observed in the 1 × dose and 100 × dose groups. During the diagnostic necropsy conducted on D6, systemic infection was indicated by the presence of these liver lesions, observed only in the 100 × dose group and in just one bird.

The most frequent pathological finding was typhlitis, indicative of *Salmonella* colonization in the gastrointestinal tract. This condition was most prevalent in the positive control and 10 × dose groups but was also observed in all other groups. Additionally, pale kidneys were observed across all groups. During the treatments, the time-trend analysis revealed that immediately following infection, all infected groups exhibited a high proportion of *Salmonella*-shedding individuals. Over the subsequent 10 days, the number of positive cases was similar among the positive control, 1 × dose, and 10 × dose groups, albeit slightly lower in the 100 × dose group. Afterward, the 1 × dose group experienced a considerably reduction in positive cases, whereas the positive control, 10 × dose, and 100 × dose groups showed greater variability but followed a similar trend. In the antibiotic-treated group, the 5-day enrofloxacin treatment (administered according to the manufacturer’s instructions) led to a considerably reduction in *Salmonella*-shedding individuals. However, subsequent observations revealed intermittent shedding, with fluctuations in the number of positive cases over time.

Alqahtani et al. (52) reported a significant increase in survival rates in mice treated with fenugreek extract following experimental infection with *Salmonella Typhimurium* compared to the control group. Similarly, Farouk et al. (53) observed reduced clinical symptoms, improved economic performance, and better histopathological outcomes in broiler chickens treated with fenugreek extract following *E. coli* infection, attributing these results to the general health-promoting effects of fenugreek.

Weight gain trends showed that up until D28, the negative control group exhibited the highest weight gain, demonstrating that the *Salmonella* infection negatively impacted weight gain in other groups. On D35, the birds in the 10 × dose group had the highest average weight. However, by D42, this difference had leveled out, with the positive control and 100 × dose-treated groups demonstrating the highest average weights. Weerasingha et al. (54) observed a growth-promoting effect when 1% fenugreek seed extract was included as a feed supplement, but higher concentrations negatively impacted growth and feed consumption. Paneru et al. (55) found no significant differences in broiler performance when 5 g/kg fenugreek supplementation was used in their diets. Moreover, Paneru et al. (42) reported that higher concentrations of fenugreek seed supplementation adversely affected bird performance.

The antimicrobial properties of fenugreek are primarily attributed to its bioactive compounds, such as saponins, alkaloids, flavonoids, and diosgenin. Saponins are known to exhibit antimicrobial activity by disrupting microbial cell membranes and interfering with bacterial adherence and colonization, potentially affecting quorum sensing mechanisms (38). Furthermore, diosgenin has been shown to inhibit bacterial growth by interfering with cell wall synthesis and disrupting biofilm formation, which may be particularly relevant in preventing pathogen colonization in the gut (39). Moreover, the antimicrobial properties of polyphenols, which are present in fenugreek, have been demonstrated against various pathogens, including *Campylobacter jejuni*, indicating their potential utility in controlling foodborne infections (56). Additionally, nanoparticles have been increasingly studied as novel antimicrobial agents that enhance bioavailability and targeting of bioactive compounds, potentially improving their efficacy (57).

The observed efficacy of the 1 × dose compared to higher concentrations may be explained by the hormesis phenomenon, where low doses of bioactive compounds exert stimulatory or protective effects, while higher doses can be detrimental (47). Additionally, the anti-quorum sensing properties of fenugreek’s compounds could reduce bacterial virulence and enhance the host’s immune response, contributing to the observed differences in weight gain and feed conversion efficiency (40).

In terms of feed consumption, the goal for producers is for birds to attain maximum weight gain with minimal feed intake. For this reason, we calculated the feed conversion ratio (FCR) for the measurable days, defined as the ratio of feed consumed to weight gained. The FCR is significantly influenced by the composition of the gut microbiome, as it plays a crucial role in nutrient breakdown. Consistently, across all time points, the group treated with the 1 × dose showed the lowest feed consumption on an individual basis, while gaining proportionally more weight. This result suggests that fenugreek at this concentration shifts the composition of the gut microbiome in a favorable direction, contributing to a more optimal balance of bacterial digestive enzymes, thereby improving not only digestion but also nutrient absorption.

This idea is supported by studies such as Khalili Samani et al. (28), who observed a positive impact of 1% fenugreek seed powder on feed consumption in laying hens. In contrast, Omri et al. (58) found no positive effect on feed consumption when using a 3% fenugreek seed-containing plant feed supplement for laying hens. Similarly, Park et al. (59) were unable to demonstrate any positive effect of fenugreek seed supplementation on feed consumption or feed conversion efficiency. This may indicate that above a certain concentration, fenugreek has a negative effect on the gut microbiome.

These seemingly contradictory findings suggest that the effects of fenugreek on gut microbiome composition and feed conversion efficiency are likely context and dose-dependent. At lower concentrations, fenugreek may act as a prebiotic, promoting beneficial bacterial growth and enhancing nutrient absorption (60). However, higher concentrations may disrupt the microbial balance, leading to reduced feed efficiency and even negative health outcomes. This dose-dependent effect aligns with the hormesis model, where low doses provide beneficial effects, but higher doses may exert detrimental outcomes (29). Further studies are needed to precisely determine the optimal dosage range that provides beneficial effects without compromising gut microbiome health or feed efficiency.

In the group treated with the 1 × dose, the pathological changes observed in deceased birds were more moderate. However, during diagnostic necropsy, the lowest frequency of pathological changes was noted in the 100 × dose compound group, which may be explained by the time elapsed between the deaths of infected birds and the diagnostic necropsy of euthanized birds. A significant reduction was observed on D12 post-infection, followed by a further decrease on D19. At the end of the trial, necropsy revealed the lowest number of major pathological changes in the group treated with the 10 × dose, while the positive control group exhibited the highest frequency. In terms of histopathological findings, the least changes were observed in birds treated with the 1 × dose, whereas the highest number of alterations occurred in the group treated with the 100 × dose.

It is noteworthy that, although resistant strains were observed at the highest rate in the 1 × dose group, this group also demonstrated the most significant reduction in *Salmonella* shedding (63% by D10, 31% by D17). Furthermore, clinical symptoms were least apparent in this group, with no diarrhea observed; only slightly softened feces were noted. Additionally, until D24, individual feed consumption was the lowest in this group, and correspondingly, the feed conversion ratio was also the most favorable up to this point.

The apparent contradiction between effective *Salmonella* shedding reduction and the higher mortality observed in the 1 × dose group may be explained by the principles of hormesis and toxicity thresholds. The 1 × dose (0.1 g/kg) appears to provide a beneficial stimulatory effect on the gut microbiome, enhancing the microbial balance and promoting efficient nutrient absorption. This could contribute to improved feed conversion efficiency and reduced *Salmonella* colonization. However, the observed mortality could be attributed to early inflammatory responses triggered by the infection, where the antimicrobial effects of the fenugreek extract may have selectively impacted certain microbial populations, leading to transient dysbiosis or metabolic stress.

Another possible explanation is that the 1 × dose, while effective in reducing bacterial colonization, may also place physiological stress on the birds due to its antimicrobial properties, especially if the dose is near the threshold where beneficial effects transition to toxic or detrimental outcomes. This dose-dependent response is consistent with the hormesis model, where low doses are beneficial, but higher doses or inappropriate timing of administration can result in adverse effects. The findings of this study are subject to certain limitations, including the controlled laboratory conditions which may not fully represent practical poultry farming environments. Additionally, the infection model using crop gavage may differ from natural exposure routes. Potential dose-dependent toxicity at higher fenugreek concentrations was not fully explored, and the relatively small sample size may have limited the statistical power of some analyses. Future studies should address these aspects to confirm the practical applicability of the results. Further studies focusing on the interactions between fenugreek supplementation, microbiome modulation, and host immune response are needed to clarify these findings.

## 5 Conclusion

This study provides valuable insights into the efficacy of different fenugreek seeds extract concentrations in mitigating the effects of Salmonella infection in chicks. Among the tested concentrations, the 1 × dose demonstrated the most favorable outcomes across multiple parameters, including reduced clinical symptoms, optimal feed conversion efficiency, and significant reductions in Salmonella shedding rates. Notably, the groups which received the 1 × dose exhibited the least apparent clinical signs of infection. They also maintained their feed consumption patterns, further supporting the potential of fenugreek extract as an effective intervention in Salmonella infections in chicks.

Our findings underscore the importance of precise dose optimization, as higher concentrations were associated with less consistent results, likely due to potential shifts in the gut microbiome. This highlights the need for further investigations, particularly metagenomic studies, to elucidate the interactions between fenugreek supplementation, gut microbiota composition, and nutrient absorption mechanisms.

In addition to identifying the optimal dose for practical applications, our study emphasizes the relevance of evaluating multiple indicators, including mortality, pathological changes, and feed conversion efficiency, to assess the broader implications of such interventions. In the future, optimizing the dosage will require not only studies on chicks but also adult hens to evaluate long-term efficacy, safety, and productivity outcomes. Moreover, detailed microbiome analysis using next-generation sequencing should be prioritized to better understand the interactions between fenugreek supplementation, gut microbiota composition, and immune response.

Future research should explore the long-term effects of fenugreek supplementation and its potential as a sustainable alternative to conventional antibiotics. Effective incorporation of fenugreek into poultry farming could enhance chick health, improve productivity, and reduce reliance on antibiotics, aligning with the principles of the One Health approach. By providing a natural, plant-based alternative that promotes gut health and reduces pathogen shedding, fenugreek supplementation holds promise for contributing to more efficient and sustainable poultry production. Additionally, lowering antibiotic use in livestock could have broader public health benefits by limiting the spread of antibiotic resistance and ensuring food safety.

## Data Availability

The original contributions presented in the study are included in the article/Supplementary material, further inquiries can be directed to the corresponding author.
