# Supplementary material for: Dose–response study of a fenugreek-based antibiotic alternative in Bábolna Tetra-SL chicks (1–42 days old) with mixed bacterial infections
Source: Front Vet Sci. 2025 Apr 24;12:1570387. doi: 10.3389/fvets.2025.1570387 (PMC12058679; doi:10.3389/fvets.2025.1570387)
Supplement: Supplementary file 1 [file Table_1.docx]

Supplementary Material

**Supplementary Table 1** The composition of the premix

| **Nutrient content** | | |
| --- | --- | --- |
| **Name** | **Value** | **Unit** |
| Dry Matter (DM) | 99.18 | % |
| Lysine | 4.53 | % |
| Methionine | 6.19 | % |
| Threonine | 3.92 | % |
| Calcium | 18.65 | % |
| Phosphorus | 7.38 | % |
| Sodium | 3.92 | % |
| Vitamin A, added | 250,000.00 | IU |
| Vitamin D3, added | 125,000.00 | IU |
| Vitamin E, added | 2 125.00 | mg |
| Choline chloride | 16,590.00 | mg |
| Niacin, added | 1 425.00 | mg |
| Pantothenic acid, added | 336.00 | mg |
| Vitamin B1, added | 90.00 | mg |
| Vitamin B12, added | 825.00 | mcg |
| Vitamin B2, added | 264.00 | mg |
| Vitamin B6, added | 144.00 | mg |
| Vitamin K3, added | 90.00 | mg |
| Biotin, added | 4 500.00 | mcg |
| Folic acid, added | 43.20 | mg |
| Zinc, added | 2 500.00 | mg |
| Iodine, added | 32.50 | mg |
| Manganese, added | 3 000.00 | mg |
| Copper, added | 387.50 | mg |
| Selenium, added | 7.50 | mg |
| Iron, added | 1 125.00 | mg |
| 6-Phytase | 13,000.00 | FTU |

**Supplementary Table 2** The groups are divided by treatment, with animals receiving pre-starter feed in weeks 1-3 and starter feed in weeks 4-6.

| **Group** | **Feed** | ***Salmonella* and *Escherichia coli* infection** | **Additive in the feed** |
| --- | --- | --- | --- |
|  |  |  |  |
| 1 | 1× dose | + | 0,1 g/kg |
| 2 |  | + |  |
| 3 |  | + |  |
| 4 | 10× dose | + | 1 g/kg |
| 5 |  | + |  |
| 6 |  | + |  |
| 7 | 100× dose | + | 10 g/kg |
| 8 |  | + |  |
| 9 |  | + |  |
| 10 | Antibiotic control | + | *Enrofloxacin |
| 11 |  | + |  |
| 12 |  | + |  |
| 13 | Positive control | + | - |
| 14 |  | + |  |
| 15 |  | + |  |
| 16 | Negative control | - | - |
| 17 |  | - |  |
| 18 |  | - |  |

* Baytril 100 mg/mL solution for mixing into drinking water A.U.V., at a dose of 10 mg/kg body weight mixed into drinking water for 5 days, according to the manufacturer's instructions.

**Supplementary Table 3** Composition of the additive in the feed.

| **Component name** | **Authorization category number** | **Authorization type** |
| --- | --- | --- |
| *Trigonella foenum graecum* extract | 2.b | Additive |
| Copper amino acid hydrate chelate | 3b406 | Additive |
| *Cichorium intybus* L. root | 4.4.1 | Raw material |
| *Curcuma longa* L.: extract | 2b | Additive |


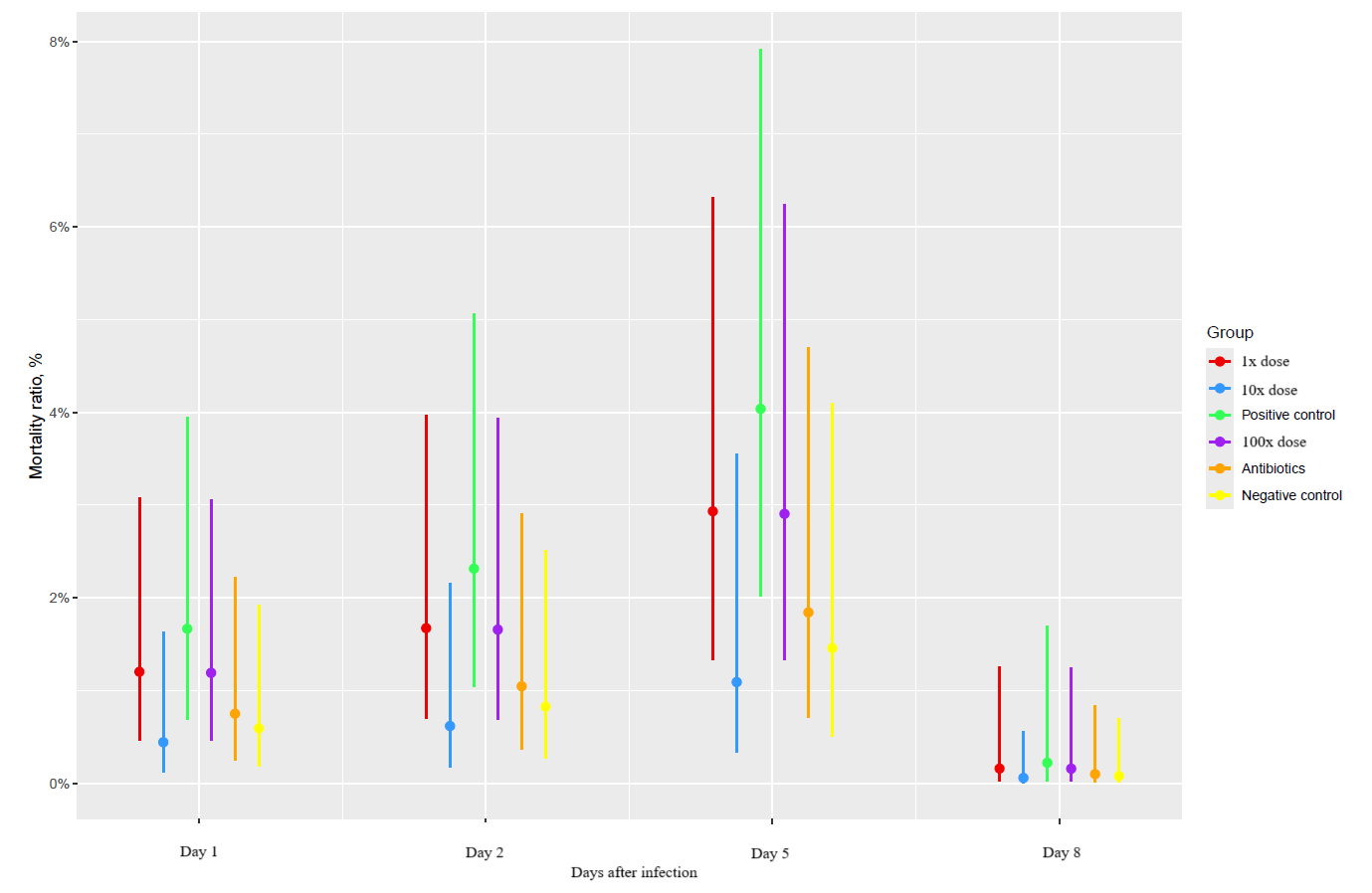


**Supplementary Figure 1** The figure below shows the model-predicted mortality rates by treatment group and date (shown as a percentage) and 95% confidence intervals.

**Supplementary Table 4** Comparison of each treatment group to a positive control using a mixed-effect logistic regression model. The dependent variable was the mortality rate, the explanatory variables were treatment group and date (without interaction), and the random effect was changing.

| **Contrast to Positive control** | **odds ratio** | | **SE** | **df** | **null** | **z.ratio** | **p.value** |
| --- | --- | --- | --- | --- | --- | --- | --- |
| 1× dose | 0.718 | 0.352 | | Inf | 1 | -0.675 | 0.8967 |
| 10×dose | 0.262 | 0.176 | | Inf | 1 | -1.998 | 0.1743 |
| 100×dose | 0.711 | 0.349 | | Inf | 1 | -0.694 | 0.8890 |
| Antibiotics | 0.446 | 0.251 | | Inf | 1 | -1.437 | 0.4579 |
| Negative control | 0.352 | 0.213 | | Inf | 1 | -1.729 | 0.2907 |

SE – standard error; df -degrees of freedom


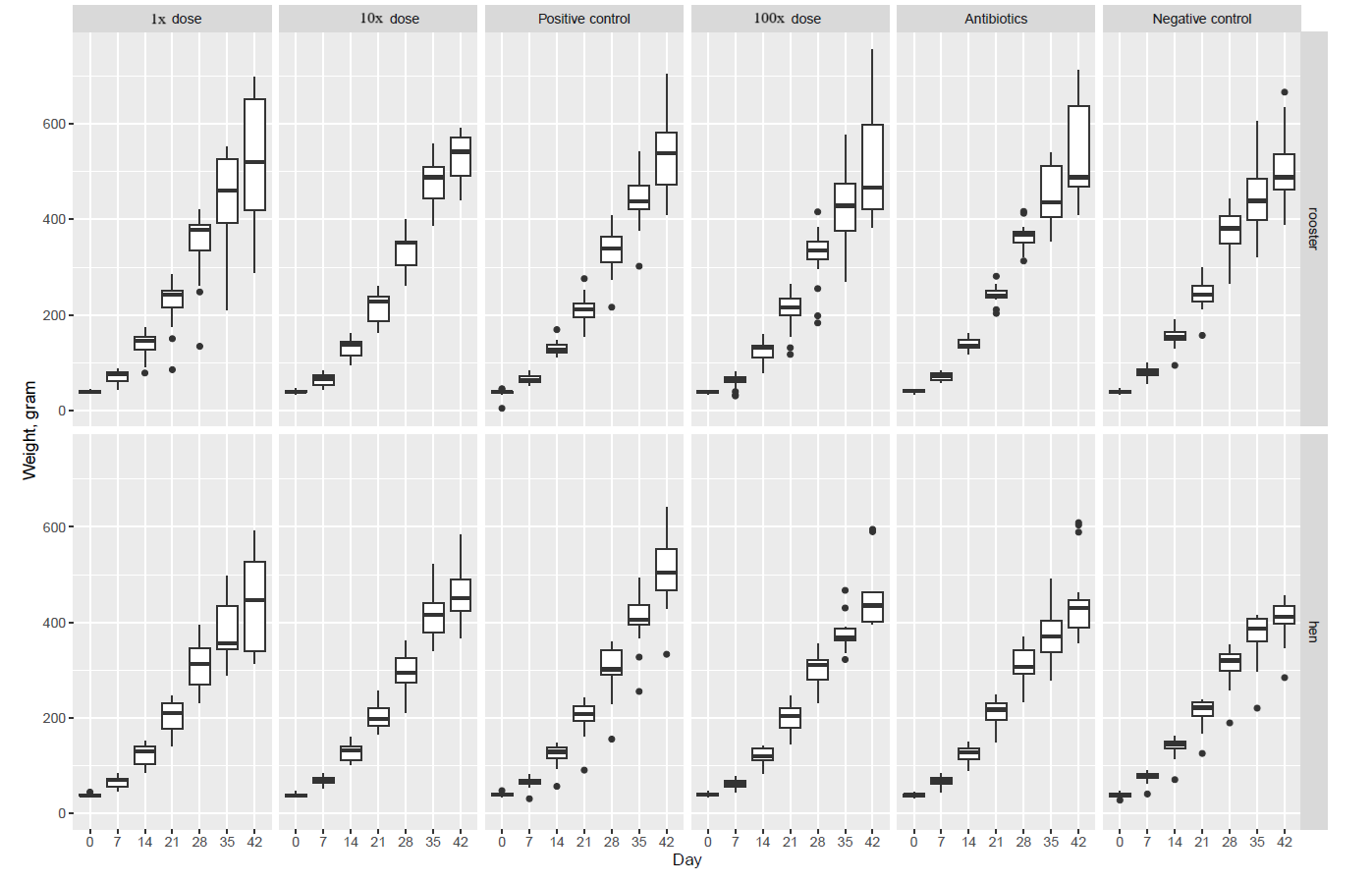


**Supplementary Figure 2** Boxplot of weight gain on each measurement day, broken down by treatment group and sex.

**Supplementary Table 5** A linear mixed model was fitted to evaluate weight gain. In the fitted model, the dependent variable was the log-transformed body weight. Fixed effects in the model included the log-transformed baseline body weight measured on day 0, the interaction of day and treatment group, and the interaction of sex and day. Individual effects (ID) were included as random effects in the model, with a random intercept structure defined to account for differences in initial body weight among individual chickens and to model the correlation arising from non-independent observations.

| **Model** | **numDF** | **denDF** | **F-value** | **p-value** |
| --- | --- | --- | --- | --- |
| (Intercept) | 1 | 918 | 220,697 | <0.0001 |
| log_weight0 | 1 | 202 | 79 | <0.0001 |
| Group | 5 | 202 | 4 | <0.0001 |
| Day | 5 | 918 | 15,050 | <0.0001 |
| Sex | 1 | 202 | 15 | <0.0001 |
| Group:Day | 25 | 918 | 9 | <0.0001 |
| Sex:Day | 5 | 918 | 13 | <0.0001 |

**Supplementary Table 6** Comparison of each treatment group to a positive control in terms of weight gain on each day of measurement.

| **Day7** | | | | | | |
| --- | --- | --- | --- | --- | --- | --- |
| **contrast to positive control** | **ratio** | **SE** | **df** | **null** | **t.ratio** | **p.value** |
| 1× dose | 1.031 | 0.0451 | 202 | 1 | 0.697 | 0.8880 |
| 10× dose | 1.011 | 0.0429 | 202 | 1 | 0.248 | 0.9925 |
| 100× dose | 0.921 | 0.0411 | 202 | 1 | -1.851 | 0.2366 |
| Antibiotics | 1.041 | 0.0460 | 202 | 1 | 0.900 | 0.7918 |
| Negative | 1.182 | 0.0517 | 202 | 1 | 3.818 | 0.0009 |
| **Day14** | | | | | | |
| **contrast to positive control** | **ratio** | **SE** | **df** | **null** | **t.ratio** | **p.value** |
| 1× dose | 1.034 | 0.0460 | 202 | 1 | 0.754 | 0.8640 |
| 10× dose | 1.017 | 0.0438 | 202 | 1 | 0.389 | 0.9752 |
| 100× dose | 0.927 | 0.0422 | 202 | 1 | -1.676 | 0.3215 |
| Antibiotics | 1.038 | 0.0466 | 202 | 1 | 0.820 | 0.8333 |
| Negative | 1.149 | 0.0512 | 202 | 1 | 3.126 | 0.0094 |
| **Day21** | | | | | | |
| **contrast to positive control** | **ratio** | **SE** | **df** | **null** | **t.ratio** | **p.value** |
| 1× dose | 1.038 | 0.0462 | 202 | 1 | 0.849 | 0.8188 |
| 10× dose | 1.024 | 0.0442 | 202 | 1 | 0.548 | 0.9390 |
| 100× dose | 0.952 | 0.0433 | 202 | 1 | -1.086 | 0.6839 |
| Antibiotics | 1.101 | 0.0495 | 202 | 1 | 2.147 | 0.1299 |
| Negative | 1.111 | 0.0495 | 202 | 1 | 2.369 | 0.0779 |
| **Day28** | | | | | | |
| **contrast to positive control** | **ratio** | **SE** | **df** | **null** | **t.ratio** | **p.value** |
| 1× dose | 1.036 | 0.0461 | 202 | 1 | 0.793 | 0.8460 |
| 10× dose | 0.996 | 0.0430 | 202 | 1 | -0.091 | 0.9995 |
| 100× dose | 0.943 | 0.0429 | 202 | 1 | -1.289 | 0.5539 |
| Antibiotics | 1.077 | 0.0484 | 202 | 1 | 1.647 | 0.3370 |
| Negative | 1.080 | 0.0481 | 202 | 1 | 1.730 | 0.2937 |
| **Day35** | | | | | | |
| **contrast to positive control** | **ratio** | **SE** | **df** | **null** | **t.ratio** | **p.value** |
| 1× dose | 0.975 | 0.0434 | 202 | 1 | -0.574 | 0.9314 |
| 10× dose | 1.044 | 0.0450 | 202 | 1 | 1.004 | 0.7335 |
| 100× dose | 0.910 | 0.0414 | 202 | 1 | -2.074 | 0.1519 |
| Antibiotics | 0.973 | 0.0438 | 202 | 1 | -0.599 | 0.9236 |
| Negative | 0.965 | 0.0429 | 202 | 1 | -0.809 | 0.8384 |
| **Day42** | | | | | | |
| **contrast to positive control** | **ratio** | **SE** | **df** | **null** | **t.ratio** | **p.value** |
| 1× dose | 0.906 | 0.0403 | 202 | 1 | -2.222 | 0.1100 |
| 10× dose | 0.943 | 0.0407 | 202 | 1 | -1.368 | 0.5034 |
| 100× dose | 0.890 | 0.0407 | 202 | 1 | -2.541 | 0.0506 |
| Antibiotics | 0.928 | 0.0417 | 202 | 1 | -1.655 | 0.3328 |
| Negative | 0.872 | 0.0388 | 202 | 1 | -3.087 | 0.0106 |

SE – standard error; df -degrees of freedom

**Supplementary Table 7** The evaluation compared the body weight trends between the cockerels and the layers on each day.

| **Day7** | | | | | | |
| --- | --- | --- | --- | --- | --- | --- |
| **contrast** | **ratio** | **SE** | **df** | **null** | **t.ratio** | **p.value** |
| rooster-hen | 1.02 | 0.0255 | 202 | 1 | 0.708 | 0.4801 |
| **Day14** | | | | | | |
| **contrast** | **ratio** | **SE** | **df** | **null** | **t.ratio** | **p.value** |
| rooster-hen | 1.07 | 0.0274 | 202 | 1 | 2.811 | 0.0054 |
| **Day21** | | | | | | |
| **contrast** | **ratio** | **SE** | **df** | **null** | **t.ratio** | **p.value** |
| rooster-hen | 1.09 | 0.0278 | 202 | 1 | 3.359 | 0.0009 |
| **Day28** | | | | | | |
| **contrast** | **ratio** | **SE** | **df** | **null** | **t.ratio** | **p.value** |
| rooster-hen | 1.12 | 0.0287 | 202 | 1 | 4.61 | <0.0001 |
| **Day35** | | | | | | |
| **contrast** | **ratio** | **SE** | **df** | **null** | **t.ratio** | **p.value** |
| rooster-hen | 1.14 | 0.0291 | 202 | 1 | 5.178 | <0.0001 |
| **Day42** | | | | | | |
| **contrast** | **ratio** | **SE** | **df** | **null** | **t.ratio** | **p.value** |
| rooster-hen | 1.15 | 0.0293 | 202 | 1 | 5.314 | <0.0001 |

SE – standard error; df -degrees of freedom

**Supplementary Table 8** Statistical analysis of feed consumption compared to positive control group.

| **contrast to positive control** | **estimate** | **SE** | **df** | **t.ratio** | **p.value** |
| --- | --- | --- | --- | --- | --- |
| 1× dose | -2.114 | 1.9 | 190 | -1.113 | 0.6672 |
| 10× dose | 3.569 | 1.9 | 190 | 1.879 | 0.2248 |
| 100× dose | -0.805 | 1.9 | 190 | -0.424 | 0.9689 |
| Antibiotics | 0.325 | 1.9 | 190 | 0.171 | 0.9972 |
| Negative | 2.554 | 1.9 | 190 | 1.344 | 0.5189 |

SE – standard error; df -degrees of freedom

**Supplementary Table 9** Statistical analysis of feed conversion ratio compared to positive control group.

| **contrast to positive control** | **estimate** | **SE** | **df** | **t.ratio** | **p.value** |
| --- | --- | --- | --- | --- | --- |
| 1× dose | -0.1784 | 0.126 | 15 | -1.412 | 0.5007 |
| 10× dose | 0.2176 | 0.126 | 15 | 1.722 | 0.3360 |
| 100× dose | -0.0199 | 0.126 | 15 | -0.158 | 0.9977 |
| Antibiotics | -0.0447 | 0.126 | 15 | -0.354 | 0.9803 |
| Negative | 0.0850 | 0.126 | 15 | 0.673 | 0.8975 |

SE – standard error; df -degrees of freedom

**Supplementary Table 10** For *Salmonella* shedding on each test day, the groups were compared to the positive control. The “ratio” represents the odds ratio and can be interpreted as follows: values close to 1 suggest that the examined group and the positive control group yield similar results, deviations from 1 indicate potential differences between the groups in terms of *Salmonella* shedding. If the ratio is less than 1, it indicates that in the positive control group, the odds of receiving a specific score versus higher scores (e.g., the odds of receiving a score of 1 versus 2 or 3) for *Salmonella* isolation is higher than in the comparable group. Conversely, if the ratio is greater than 1, it suggests that the odds of specific score versus higher scores are higher in the comparable group than in the positive control group.

| **Day2** | | | | | | |
| --- | --- | --- | --- | --- | --- | --- |
| **contrast to positive control** | **ratio** | **SE** | **df** | **null** | **z.ratio** | **p.value** |
| 1× dose | 0.57 | 0.2 | Inf | 1 | -1.732 | 0.2891 |
| 10× dose | 0.42 | 0.2 | Inf | 1 | -2.389 | 0.0709 |
| 100× dose | 0.56 | 0.2 | Inf | 1 | -1.627 | 0.3447 |
| Antibiotics | 0.69 | 0.2 | Inf | 1 | -1.019 | 0.7237 |
| Negative | 313.34 | 224.4 | Inf | 1 | 8.025 | <0.0001 |
| **Day5** | | | | | | |
| **contrast to positive control** | **ratio** | **SE** | **df** | **null** | **z.ratio** | **p.value** |
| 1× dose | 0.92 | 0.4 | Inf | 1 | -0.198 | 0.9959 |
| 10× dose | 0.68 | 0.3 | Inf | 1 | -0.909 | 0.7870 |
| 100× dose | 1.95 | 0.9 | Inf | 1 | 1.510 | 0.4132 |
| Antibiotics | 17.03 | 8.1 | Inf | 1 | 5.968 | <0.0001 |
| Negative | 1100.91 | 1415 | Inf | 1 | 5.449 | <0.0001 |
| **Day7** | | | | | | |
| **contrast to positive control** | **ratio** | **SE** | **df** | **null** | **z.ratio** | **p.value** |
| 1× dose | 0.40 | 0.2 | Inf | 1 | -2.245 | 0.1009 |
| 10× dose | 0.41 | 0.2 | Inf | 1 | -2.122 | 0.1336 |
| 100× dose | 1.25 | 0.5 | Inf | 1 | 0.516 | 0.9479 |
| Antibiotics | 5.27 | 2.5 | Inf | 1 | 3.556 | 0.0018 |
| Negative | 488.68 | 623 | Inf | 1 | 4.857 | <0.0001 |
| **Day9** | | | | | | |
| **contrast to positive control** | **ratio** | **SE** | **df** | **null** | **z.ratio** | **p.value** |
| 1× dose | 0.50 | 0.2 | Inf | 1 | -1.720 | 0.2956 |
| 10× dose | 0.62 | 0.3 | Inf | 1 | -1.156 | 0.6388 |
| 100× dose | 0.95 | 0.4 | Inf | 1 | -0.107 | 0.9992 |
| Antibiotics | 2.75 | 1.2 | Inf | 1 | 2.232 | 0.1040 |
| Negative | 523.61 | 696.6 | Inf | 1 | 4.706 | <0.0001 |
| **Day12** | | | | | | |
| **contrast to positive control** | **ratio** | **SE** | **df** | **null** | **z.ratio** | **p.value** |
| 1× dose | 0.92 | 0.4 | Inf | 1 | -0.208 | 0.9954 |
| 10× dose | 0.85 | 0.4 | Inf | 1 | -0.400 | 0.9733 |
| 100× dose | 0.86 | 0.4 | Inf | 1 | -0.349 | 0.9814 |
| Antibiotics | 0.34 | 0.2 | Inf | 1 | -2.442 | 0.0619 |
| Negative | 820.66 | 1155.6 | Inf | 1 | 4.765 | <0.0001 |
| **Day19** | | | | | | |
| **contrast to positive control** | **ratio** | **SE** | **df** | **null** | **z.ratio** | **p.value** |
| 1× dose | 1.87 | 0.8 | Inf | 1 | 1.433 | 0.4601 |
| 10× dose | 1.53 | 0.7 | Inf | 1 | 0.962 | 0.7575 |
| 100× dose | 1.35 | 0.6 | Inf | 1 | 0.641 | 0.9093 |
| Antibiotics | 1.15 | 0.5 | Inf | 1 | 0.316 | 0.9857 |
| Negative | 1211.15 | 1941.2 | Inf | 1 | 4.429 | <0.0001 |
| **Day26** | | | | | | |
| **contrast to positive control** | **ratio** | **SE** | **df** | **null** | **z.ratio** | **p.value** |
| 1× dose | 2.26 | 1.0 | Inf | 1 | 1.840 | 0.2379 |
| 10× dose | 1.06 | 0.5 | Inf | 1 | 0.144 | 0.9982 |
| 100× dose | 1.17 | 0.5 | Inf | 1 | 0.335 | 0.9833 |
| Antibiotics | 1.76 | 0.8 | Inf | 1 | 1.219 | 0.5980 |
| Negative | 1423.25 | 2311.8 | Inf | 1 | 4.470 | <0.0001 |
| **Day33** | | | | | | |
| **contrast to positive control** | **ratio** | **SE** | **df** | **null** | **z.ratio** | **p.value** |
| 1× dose | 4.61 | 2.1 | Inf | 1 | 3.412 | 0.0031 |
| 10× dose | 1.22 | 0.5 | Inf | 1 | 0.455 | 0.9625 |
| 100× dose | 1.72 | 0.8 | Inf | 1 | 1.190 | 0.6168 |
| Antibiotics | 1.26 | 0.6 | Inf | 1 | 0.527 | 0.9451 |
| Negative | 2852.77 | 4693.9 | Inf | 1 | 4.835 | <0.0001 |
| **Day40** | | | | | | |
| **contrast to positive control** | **ratio** | **SE** | **df** | **null** | **z.ratio** | **p.value** |
| 1× dose | 1.40 | 0.6 | Inf | 1 | 0.747 | 0.8670 |
| 10× dose | 1.61 | 0.7 | Inf | 1 | 1.039 | 0.7116 |
| 100× dose | 1.18 | 0.6 | Inf | 1 | 0.354 | 0.9807 |
| Antibiotics | 0.83 | 0.4 | Inf | 1 | -0.390 | 0.9750 |
| Negative | 881.47 | 1429.3 | Inf | 1 | 4.182 | 0.0001 |

SE – standard error; df -degrees of freedom
